# Njaoaminiums A, B, and C: Cyclic 3-Alkylpyridinium Salts from the Marine Sponge *Reniera* sp

**DOI:** 10.3390/molecules14114716

**Published:** 2009-11-19

**Authors:** Remi Laville, Grégory Genta-Jouve, Carlos Urda, Rogelio Fernández, Olivier P. Thomas, Fernando Reyes, Philippe Amade

**Affiliations:** 1 Laboratoire de Chimie des Molécules Bioactives et des Arômes, Institut de Chimie de Nice, Faculté des Science, Université de Nice-Sophia Antipolis, UMR 6001 CNRS, Parc Valrose, 06108 Nice Cedex 02, France; 2 Medicinal Chemistry Department, PharmaMar S.A., Pol. Ind. La Mina Norte, Avenida de los Reyes 1, 28770 (Colmenar Viejo) Madrid, Spain

**Keywords:** marine natural products, marine sponge, *Reniera*, alkylpyridinium, circular dichroism, absolute configuration

## Abstract

Three novel cyclic 3-alkylpyridinium salts, named njaoaminiums A, B, and C (**1-3**), were isolated from the marine sponge *Reniera* sp*.,* collected off the coasts of Pemba Island, Tanzania. The structural determination of the compounds was based on 1D and 2D NMR studies and mass spectral determinations. Njaoaminiums B (**2**) and C (**3**) are the first examples of cyclic 3-alkylpyridinium salts bearing a methyl substituent on the alkyl chains. These compounds are assumed to be biosynthetic precursors of the njaoamines, previously isolated from the same sponge. The absolute configurations of the methyls of **2** and **3** were assigned by comparison between experimental and TDDFT calculated circular dichroism spectra on the most stable conformer. Compound **2** showed weak cytotoxicity against the three human tumor cell lines MDA-MB-231, A549, and HT29.

## Introduction

3-Alkylpyridinium salts form a large class of natural products widely distributed in marine sponges of the order Haplosclerida [1,2]. Monomeric, dimeric, trimeric, as well as polymeric 3-alkylpyridinium salts are known to exhibit a wide range of biological activities, including cytotoxicity and ichthyotoxicity [3]. Common representatives of this family are the cyclostellettamines [4,5], viscosamine [6], or the cyclohaliclonamines [7]. Cyclic 3-alkylpyridinium salts have been proposed as biogenetic precursors of important classes of polycyclic alkaloids such as the ingenamine [8],keramaphidin [9], xestocyclamine [10], halicyclamine [11], or manzamine families [12,13].

Chemical investigation of a marine sponge *Reniera* sp. collected off the coast of Tanzania previously led to the isolation of a new family of compounds, the njaoamines A-D [14]. As products of a transannular cycloaddition of bis-3-alkylpyridinium salts, they are assumed to be biogenetically linked to the ingenamine family. In order to obtain additional information about the biosynthesis of these intriguing compounds, we looked further into the minor constituents of this sponge. We report herein the structural determination of three new bis-3-alkylpyridinium salts named njaoaminiums A, B, and C (**1**-**3**), that are putative precursors of the njaoamine family.

## Results and Discussion

**Figure 1:**
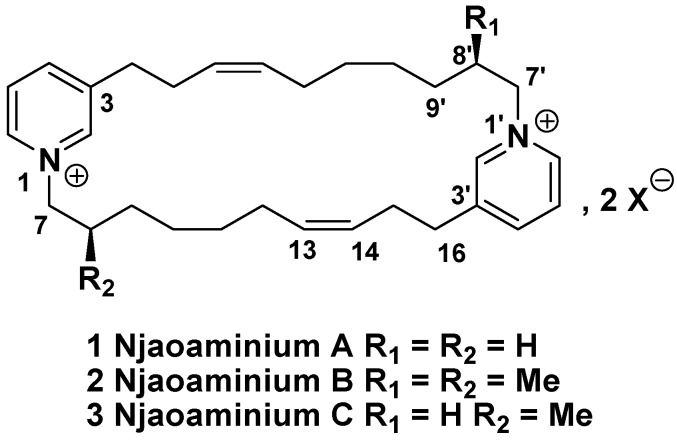
Structure of njaoaminiums A, B, and C (1-3).

The 2-propanol crude extract of *Reniera* sp. was dissolved in 4:1 H_2_O/MeOH and subjected to a solvent-solvent partition with *n*-hexane, AcOEt, and *n*-BuOH. The butanol extract was fractionated by Vacuum Liquid Chromatography, eluting with a gradient of decreasing polarity from H_2_O to MeOH. The subsequent H_2_O/MeOH 1:1 fraction was purified by semi-preparative reverse-phase HPLC (Symmetry C_18 _column, Waters, 7.8 x 150 mm, gradient H_2_O/MeCN/TFA 80:20:0.1 to 50:50:0.1) to afford pure compounds **1** (4.0 mg), **2** (4.8 mg), and **3** (4.2 mg) (Figure 1).

Njaoaminium A (**1**) was isolated as a colorless oil and its molecular formula C_30_H_44_N_2_^2+^, was deduced from HRESIMS (*m*/*z* 545.3323 [M+TFA^-^]^+^, Δ -4.8 ppm). The ^1^H-NMR spectrum of compound **1** exhibited signals characteristic of a 3-alkylpyridinium moiety, including aromatic hydrogen signals at *δ*_H_ 8.87 (s, 2H, H-2), 8.81 (d, *J* = 6.0 Hz, 2H, H-6), 8.41 (d, *J* = 8.0 Hz, 2H, H-4), 8.00 (dd, *J* = 8.0, 6.0 Hz, 2H, H-5) ppm as well as α-hydrogen signals at *δ*_H_ 4.58 (t, *J* = 7.0 Hz, 4H, H-7), 2.96 (t, *J* = 6.5 Hz, 4H, H-16) ppm (Table 1).The UV absorbance maximum at *λ*_max_ 267 nm corroborated this conclusion. Because two alkenes were evidenced by the signals at *δ*_H_ 5.40 (m, 4H, H-13 and H-14) ppm and *δ*_C_ 127.8 (CH, C-14), 133.4 (CH, C-13) ppm, we first thought that compound **1** would be identical to cyclohaliclonamine A [15]. However, the key H-16/H-15/H-14/H-13 COSY correlations unequivocally placed the unsaturations at the C-13/C-14 position instead of the C-9/C-10 position of the cyclohaliclonamines. Confirmation was given by the key H-16/C-14, H-15/C-14, and H-15/C-13 HMBC correlations. The configuration of the C=C double bond was assigned to be *Z* because of the shielding of the α-carbons of the double bond: *δ*_C_ 28.1 (CH_2_, C-12), 29.3 (CH_2_, C-15) ppm instead of the values greater than 35 ppm for an *E* configuration [15]. The (+)-ESIMS/MS data were used to confirm the symmetry of the molecule. Indeed the only observed MS^2^ fragment at *m*/*z* 216.2 was assigned to a Hoffmann-type fragmentation at the N-1/C-7 position of the pyridiniums.

**Table 1:** ^1^H- (400 MHz, CD_3_OD) and ^13^C- (100 MHz, CD_3_OD) NMR data of compounds **1**-**3**.

	1		2			3
no.	*δ*_C_	*δ*_H_ m (*J* in Hz)	*δ*_C_	*δ*_H_ m (*J* in Hz)	*δ*_C_	*δ*_H_ m (*J* in Hz)
2	145.6	8.87 s	146.0	8.83 s	145.6	8.85 s
2’					145.7	8.83 s
3	144.9		144.9		144.9	
3’					145.0	
4	147.4	8.41 d (8.0)	147.4	8.46 d (8.0)	147.3	8.42 d (8.0)
4’					147.4	8.44 d (8.0)
5	128.8	8.00 dd (8.0, 6.0)	128.8	8.02 dd (8.0, 6.0)	128.7	7.99 m
					128.9	8.01 m
6	143.3	8.81 d (6.0)	143.8	8.81 d (6.0)	143.4	8.81 d (6.0)
6’					143.8	8.79 d (6.0)
7	62.9	4.58 t (7.0)	68.3	4.53 dd (12.5, 5.5)	62.9	4.58 t (7.0)
				4.34 dd (13.0, 9.0)		
7’					68.3	4.49 dd (12.5, 5.5)
						4.35 dd (13.0, 9.0)
8	32.9	1.92 quint (7.0)	36.7	2.03 m	32.6	1.91 m
8’					36.7	2.02 m
9	27.1	1.25 m	33.9	1.11 m	27.0	1.25 m
9’					34.0	1.11 m
10	30.2	1.16 m	27.4	1.30 m	30.0	1.16 m
10’					27.4	1.30 m
11	30.4	1.10 m	30.6	1.10 m	30.4	1.10 m
11’					30.7	1.10 m
12	28.1	1.60 q (7.0)	28.1	1.71 q (7.0)	28.1	1.63 m
12’					28.1	1.69 m
13	133.4	5.40 m	133.3	5.38 m	133.3	5.38 m
13’					133.2	5.38 m
14-14’	127.8	5.40 m	128.1	5.38 m	128.0	5.38 m
15	29.3	2.46 q (6.5)	29.3	2.46 q (6.5)	29.3	2.45 m
16	33.1	2.96 t (6.5)	33.0	2.96 t (6.5)	33.1	2.95 t (6.5)
Me			16.9	1.01 d (7.0)	16.9	0.98 d (7.0)

**Figure 2:**
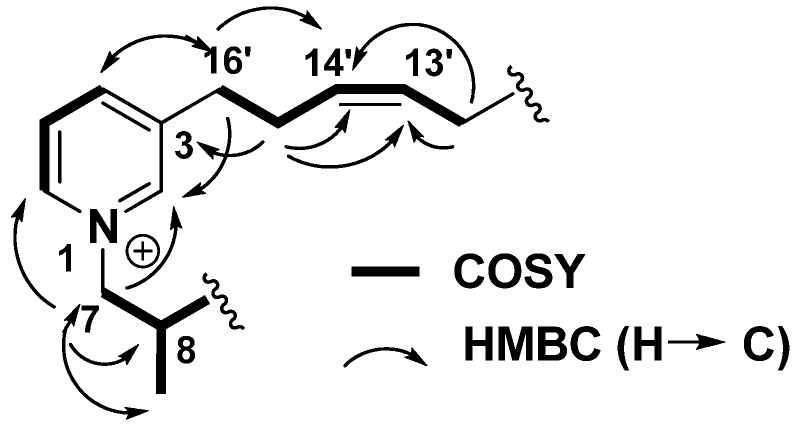
Key COSY and HMBC correlations of njaoaminium B (**2**).

Njaoaminium B (**2**) was isolated as a colorless oil and its molecular formula C_32_H_48_N_2_^2+^, was deduced from HRESIMS (*m*/*z* 573.3642 [M+TFA^-^]^+^, Δ 3.5 ppm). Comparing to the molecular formula of **1**, two additional methylenes were therefore present and the C2 symmetry of the molecule was still evidenced in the ^1^H-NMR spectrum. In this spectrum, the presence of a doublet at *δ*_H_ 1.01 (*J* = 7.0 Hz, 6H, H-Me) ppm suggested the presence of two methyl groups placed on the cyclic alkyl chain. The H-Me/H-8/H-7 COSY correlations allowed location of these methyl groups at the C-8 positions (Figure 2).

Njaoaminium C (**3**) was isolated as a colorless oil and its molecular formula C_31_H_46_N_2_^2+^, was deduced from HRESIMS (*m*/*z* 559.3519 [M+TFA^-^]^+^, Δ 2.3 ppm). The ^1^H- and ^13^C-NMR spectra were almost superimposable on the spectra of **1** and **2**. The presence of both fragments at *m*/*z* 216.2 and 230.2 in the ESIMS-MS spectrum of **3** confirmed that **3** possessed only one methyl group at C-8. The C2 symmetry, present in **1** and **2**, was thus absent in **3**.

**Scheme 1:**
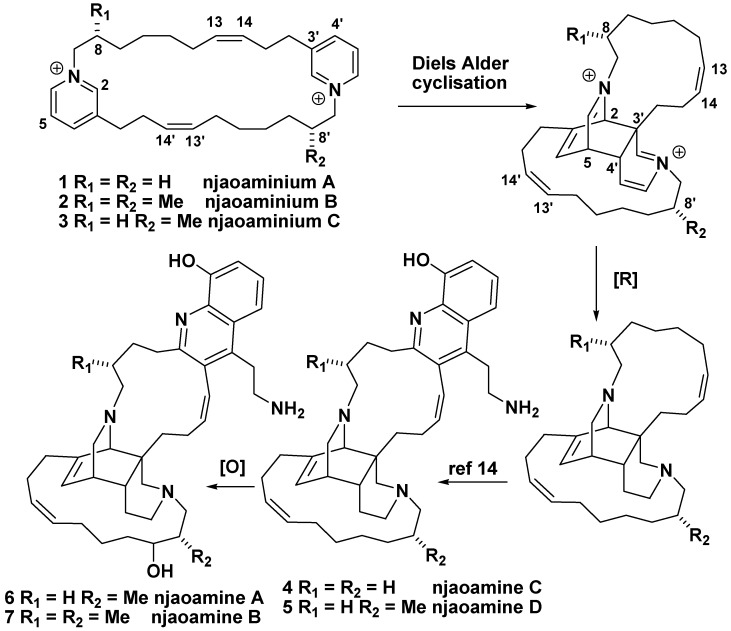
Biosynthetic hypotheses linking njaoamines A-D (**4-7**) to njaoaminiums A-C (**1-3**).

**Figure 3:**
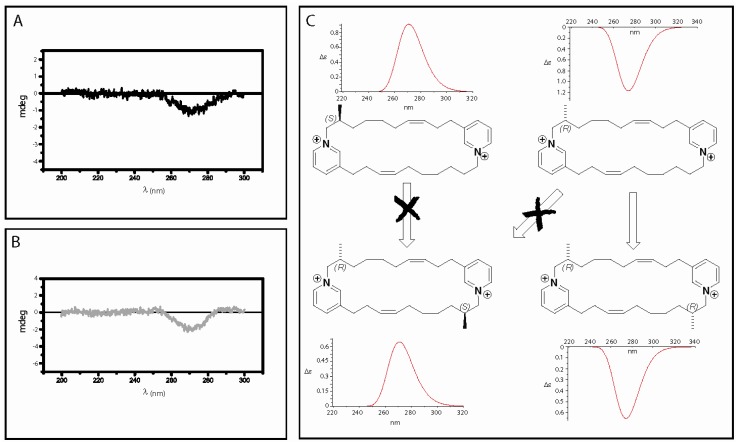
**A**. Experimental CD spectrum of **3**; **B**. Experimental CD spectrum of **2**; C. Calculated CD spectra of (*S*)-**3**, (*R*)-**3**, (*R,R*)-**2**, and (*S,R*)-**2** in MeOH (moved 57 nm left).

Because the absolute configurations of the methyl substituents present in the njaoamines were not elucidated, we used circular dichroism on njaoaminiums B (**2**) and C (**3**) to gain some information to this end. Unfortunately no exciton effect between both pyridiniums was observed in the CD spectra of **2** and **3,** which did not allow us to reach any conclusions. We therefore decided to compare the experimental CD spectrum of **3** in MeOH with the calculated ones after energy minimisation with the DFT method at B3LYP/6-31G level in MeOH. A negative Cotton effect was observed in the experimental CD spectrum of **3** at 267 nm which fits with the electronic transition observed in the UV spectrum at this wavelength (π→π* pyridinium transition). Bands of opposite signs were obtained by TDDFT CD calculations on the lowest energy conformers (Boltzmann weighted ΔE < 2.5 kcal.mol^-1^) of both enantiomers: positive for the *S* configuration, and negative for the *R* configuration (Figure 3). Even if the molecules seem highly flexible only small changes in the 3D structure of the compounds were observed and we assumed that they could not induce a change in the sign of the calculated Cotton effect. The absolute configuration of the methyl at C-8 was then assigned as *R* for compound **3**. For compound **2**, two reasons led us to assume a (*R*,*R*) configuration. First, comparison between their ^1^H-NMR spectra seemed to indicate the C2 symmetry was conserved between compounds **1** and **2**. Then, the sign of the unique Cotton effect was still negative for **2**. Furthermore, in the case of a (*R*,*S*) configuration the inversion center would have rendered the molecule achiral. Using TDDFT calculations we could confirm that this result was only in accordance with a (*R*,*R*) configuration (Figure 3). Due to our biosynthetic hypotheses these results suggested the same absolute configurations for the methyls of the previously isolated njaoamines (Scheme 1).

Compounds **1**-**3** were tested against three human tumor cell lines, including A549 (lung carcinoma), HT29 (colon carcinoma), and MDA-MB-231 (breast). Only compound **2** showed cytotoxicity at concentrations below 10 µΜ, with GI_50_ values of 4.1 (A549), 4.2 (HT29), and 4.8 (MDA-MB-231) μM [16].

## Experimental

### General

Optical rotations were measured on a Jasco P-1020 polarimeter. UV and CD spectra were measured using a JASCO J-810 spectropolarimeter. IR spectra were recorded on a Perkin-Elmer Paragon 1000 FT-IR spectrophotometer. NMR experiments were performed on a Varian Unity 500 spectrometer at 500/125 MHz (^1^H/^13^C). Chemical shifts were reported in ppm using residual CD_3_OD (*δ* 3.31 for ^1^H and 49.0 for ^13^C) as internal reference. ESIMS/MS were performed on a Bruker Esquire 3000 Plus spectrometer (Ion-Trap). HRESIMS were performed on a QStar Applied Biosystems spectrometer.

### Animal material

The sponge specimens of *Reniera* sp. were collected in December 2004 by hand using SCUBA at Njao (Pemba island, Tanzania), at depths ranging from 7 to 38 m, and kept frozen until used. The material was identified by Dr. José Luis Carballo from the Universidad Autónoma de México (México). A voucher specimen (ORMA033212) is deposited at PharmaMar (Spain).

### Extraction and isolation

Frozen specimens of the sponge (803 g) were triturated and exhaustively extracted with 2-propanol (3 × 1 L). The extract was concentrated under vacuum to yield 84 g of crude extract. This crude material was dissolved in 4:1 H_2_O/MeOH (500 mL) and partitioned between *n*-hexane (3 × 500 mL), EtOAc (3 × 500 mL) and *n*-BuOH (2 × 500 mL). The *n*-BuOH extract (8.2 g) was subjected to reversed phase VLC over RP-18 silica gel with a step gradient from H_2_O to MeOH. Fractions eluted with MeOH/H_2_O 1:1 were subjected to semipreparative HPLC (Symmetry C_18_ column, Waters, 7.8 × 150 mm, gradient H_2_O/MeCN/TFA 80:20:0.1 to 50:50:0.1 in 40 min) to yield compounds **1** (4.0 mg), **2** (4.2 mg), and **3** (4.8 mg).

### Characterization data

*Njaoaminium A* (**1**): Colorless oil; UV (MeOH) *λ*_max_ 267 nm (log ε 3.7); IR *ν*_max_ (neat) 2934, 2865, 1678, 1450, 1199, 1125 cm^-1^; ^1^H-NMR, see Table 1; ^13^C-NMR, see Table 1; ESIMS *m*/*z* 545.2 [M+TFA^-^]^+^; HRESIMS *m*/*z* 545.3323 [M+TFA^-^]^+^, Δ -4.8 ppm.

*Njaoaminium B* (**2**): Colorless oil; [α]^24^_D_ -9.4° (*c* 0.108, MeOH); UV (MeOH) 267 nm (log ε 3.7); CD (MeOH, *c* 3x10^-4^ M) *Δε* (*λ*_max_ nm) -0.22 (267); IR *ν*_max_ (neat) 2940, 2865, 1675, 1458, 1199, 1132 cm^-1^; ^1^H-NMR, see Table 1; ^13^C-NMR, see Table 1; ESIMS *m*/*z* 573.2 [M+TFA^-^]^+^; HRESIMS *m*/*z* 573.3642 [M+TFA^-^]^+^, Δ 3.5 ppm.

*Njaoaminium C* (**3**): Colorless oil; [α]^24^_D_ -13.3° (*c* 0.086, MeOH); UV (MeOH) 267 nm (log ε 3.7); CD (MeOH, *c* 3x10^-4^ M) *Δε* (*λ*_max_ nm) -0.12 (267); IR *ν*_max_ (neat) 2938, 2857, 1678, 1445, 1199, 1127 cm^-1^; ^1^H-NMR, see Table 1; ^13^C-NMR, see Table 1; ESIMS *m*/*z* 559.2 [M+TFA^-^]^+^; HRESIMS *m*/*z* 559.3519 [M+TFA^-^]^+^, Δ 2.3 ppm.

### Calculations

All the calculations were performed at 298 K by the Gaussian03 program package [15]. The Density Functional Theory (DFT) was used to scan the potential energy surface (PES) at the B3LYP/6-31G level to identify the most stable conformer. The Integral Equation Formalism Polarized Continuum Model (IEFPCM) was used for solvation. Ground-state geometries were optimized at the B3LYP/6-31G level. TDDFT was employed to calculate excitation energy (in eV) and rotatory strength *R* in dipole velocity (*R*_vel_) and dipole length (*R*_len_) forms. The calculated rotatory strengths were simulated in ECD curve by using the Gaussian function:
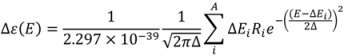
(1)
where Δ is half the width of the band at 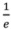 height and Δ*E_i_* and *R_i_* are the excitation energies and the rotatory strengths for transition *i*, respectively, Δ = 0.20 eV and *R_vel_* were used.

### Biological activity

A colorimetric assay using sulforhodamine B has been adapted for a quantitative measurement of cell growth and viability following the technique described in the literature [15]. The *in vitro* activity of the compounds was evaluated against three tumor cell lines, including lung carcinoma A 549, colon carcinoma HT29, and breast MDA-MB-231.

## Conclusions

Three new cyclic 3-alkylpyridinium salts have been isolated from the marine sponge *Reniera* sp. Njaoaminiums B (**2**) and C (**3**) are the first examples of cyclic 3-alkylpyridinium salts bearing methyl substituents on the alkyl chain. The biosynthetic origin of these methyls may arise from a propionate unit incorporated at the C-7 position. These compounds are assumed to be biosynthetic precursors of the njaoamines A-D (**4**-**7**) after transannular cycloaddition of the pyridinium moieties of compounds **1**-**3**, subsequent reduction of three double bonds, and fixation of the quinoline moiety.
